# Phase I study of oral azacitidine plus salvage chemotherapy in relapsed/refractory diffuse large B-cell lymphoma

**DOI:** 10.1007/s00277-026-07125-7

**Published:** 2026-06-22

**Authors:** Brian Hess, Nina D. Wagner-Johnston, Lindsey Hendrickson, James A. Davis, Elizabeth Hill, Anshu Giri, Kent Armeson, Maria V. Revuelta, Shanta Salzer, Robin Klingenberg, Leandro Cerchietti

**Affiliations:** 1 Hollings Cancer Center, The Medical University of South Carolina, Charleston, SC USA; 2 Sidney Kimmel Comprehensive Cancer Center, Johns Hopkins University, Baltimore, MD USA; 3 Department of Hematology/Oncology, Temple-Fox Chase Cancer Center, Philadelphia, PA USA; 4 Laura and Isaac Perlmutter Cancer Center, NYU Langone Health, New York University, New York, NY USA

**Keywords:** Lymphoma, Dlbcl, Epigenetics

## Abstract

Salvage chemotherapy and autologous stem cell transplantation (ASCT) offer the opportunity to cure eligible patients with relapsed diffuse large B-cell lymphoma (DLBCL). Epigenetic alterations such as aberrant DNA methylation patterns have been linked to chemotherapy resistance in DLBCL. Oral Azacitadine (AZA) is a hypomethylating agent that inhibits DNA methyltransferase and has provided evidence of chemotherapy sensitization in DLBCL. In this phase I trial the safety and feasibility of two dose levels of AZA were investigated in combination with standard cytotoxic chemotherapy rituximab, ifosfamide, carboplatin, and etoposide (R-ICE) in relapsed DLBCL patients who were candidates for ASCT.

**Supplementary Information:**

The online version contains supplementary material available at https://doi.org/10.1007/s00277-026-07125-7.

## Introduction

Approximately 40% of patients with diffuse large B-cell lymphoma (DLBCL) experience relapsed or refractory (R/R) disease. For those not meeting an indication for second-line CAR T-cell therapy, salvage chemotherapy followed by autologous stem cell transplant (ASCT) remains the standard of care for transplant eligible patients, though success is contingent upon tumor response to salvage chemotherapy [1–4]. Aberrant DNA methylation has emerged as a key driver of chemotherapy resistance and immune evasion in DLBCL, characterized by the silencing of tumor suppressor genes and the depletion of the lymphoma microenvironment (LME), characterized by a lack of immune infiltration [5–9]. DLBCL cases with a depleted LME, common in relapsed DLBCL, are also characterized by increased DNA hypermethylation [10]. Preclinical data suggest that hypomethylating agents like oral azacitidine (AZA) can reverse these epigenetic alterations, sensitizing resistant cell populations to cytotoxic therapy [10–12]. Based on this rationale, we conducted a phase I trial to evaluate the safety and feasibility of combining AZA with standard R-ICE (rituximab, ifosfamide, carboplatin, etoposide) salvage therapy in transplant-eligible R/R DLBCL.

## Methods

This was an open-label phase I study conducted at the Hollings Cancer Center at the Medical University of South Carolina and Sidney Kimmel Cancer Center at Johns Hopkins University Center (NCT02343536). This study was approved by the respective institutional review boards at each institution. Written informed consent was obtained from each patient, and research conducted in accordance with the Declaration of Helsinki. Eligible participants were adults (≥ 18 years) with relapsed/refractory (R/R) DLBCL, grade 3B follicular lymphoma, or transformed indolent lymphoma who were candidates for ASCT. All patients received ≥ 1 prior anti-CD20 multi-agent regimen and had measurable disease by Lugano 2014 criteria [13]. The protocol was amended post-activation to exclude patients > 70 years with ECOG PS > 1 due to toxicity concerns.

**Fig. 1:**
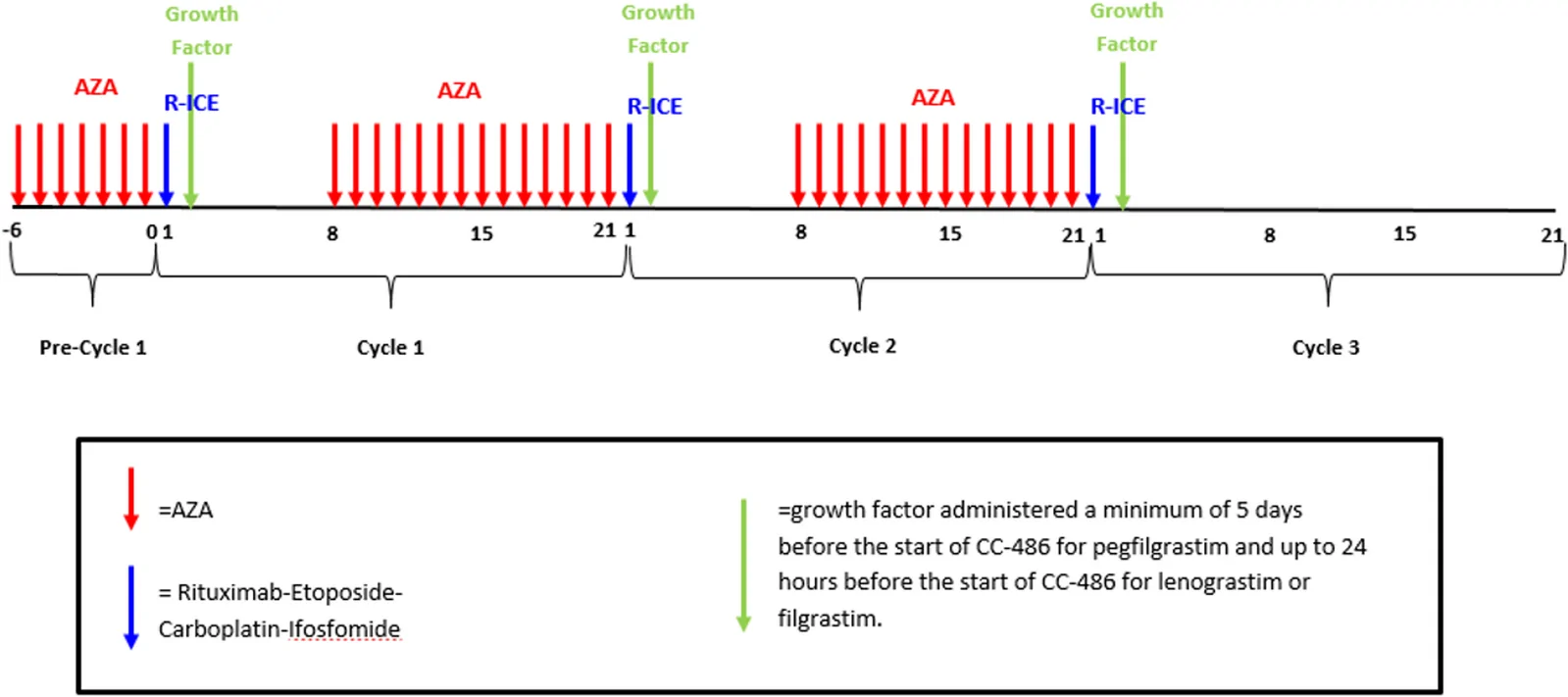
Dosing schedule

Patients received standard dosing of R-ICE chemotherapy as follows: rituximab (375 mg/m² day 1), ifosfamide (5,000 mg/m² day 2), carboplatin (AUC 5 day 2), and etoposide (100 mg/m² days 1–3) (R-ICE). Azacitidine (AZA) was administered (days − 6 to 0 prior to cycle 1; days 8–21 of cycles 1–2) (Fig. 1). Each cycle was 21-days. Dose escalation followed a standard 3 + 3 design at 200 mg (DL1), 300 mg (DL2), and 150 mg (DL-1). Dose modifications of any of the components of R-ICE chemotherapy due to hematologic or non-hematologic toxicity were allowed per institutional guidelines and according to each package insert. Dose modifications and delays of CC-486 were followed as described in Appendix Tables 1 and 2. Dose-limiting toxicity (DLT) was defined by CTCAE v5.0 as a Grade ≥ 3 adverse event leading to a > 7-day cycle delay or Grade ≥ 2 vomiting/diarrhea persisting > 48 h despite best supportive care. The DLT period included day − 6 of cycle 1 to day 1 of cycle 2. The trial explored two dose cohorts of CC-486 following a standard 3 + 3 dose-escalation design. DL1 was 200 mg, 100 mg lower than the CC-486 RP2D used in combination with R-CHOP chemotherapy in the phase I trial (NCT02343536) and DL2 was 300 mg.

**Table 1:** Baseline characteristics

Characteristic	*N* = 9 (%)
Gender	
Female	4 (44)
Male	5 (56)
Histology	
DLBCL, NOS	1 (11)
DLBCL, GCB subtype	1 (11)
DLBCL, non-GCB subtype	2 (22)
THRLBCL	2 (22)
HGBCL	1 (11)
Transformed from Indolent Lymphoma	2 (22)
Age	
Median (range)	58 (45–71)
Relapse After Frontline Chemotherapy	
< 12 months	8 (89)
* ≥* 12 months	1 (11)

*DLBCL* diffuse large B-cell lymphoma, *NOS* not otherwise specified, *GCB* germinal center B-cell subtype, *THRLBCL* T-cell/histiocyte-rich large B-cell lymphoma, *HGBCL* high-grade B-cell lymphoma

**Table 2:** Dose delays, missed doses, and adverse events by dose level

Dose Level (*N*)	DL1–200 mg (6)	DL2- 300 mg (3)	Combined (9)
Missed Doses of AZA due to Cytopenias (%)			
Pre-phase	0 (0)	4 (19)	4 (6)
Cycle 1	0 (0)	19 (45)	19 (15)
Cycle 2	7 (8)	9 (32)	16 (14)
Missed/Planned Total	7/210 (4)	32/91 (35)	39/301 (13)
Cycle 2 or 3 R-ICE chemotherapy delay (%)	0/12 (0)	4/5 (80)	4/17 (24)
Completion of 3 cycles of R-ICE (%)	6 (100)	2 (67)	8 (89)
Grade ≥ 3 Adverse Events			
Related to AZA	5	15	20
Hematologic	5	8	13
Neutropenic Fever/Cycles (%)	0/18 (0)	2/7 (28)	2/25 (8)
Dose Limiting Toxicity (%)	0 (0)	1 (33)	1 (11)

The primary objective was to determine the recommended phase 2 dose (RP2D). Secondary endpoints included evaluation of overall response rate (ORR) and complete remission (CR) rate after completion of study protocol therapy per 2014 International Working Group (IWG) criteria, stem cell mobilization feasibility, and proportion of patients proceeding to ASCT. Safety was monitored weekly via hematology assessments; response was assessed by PET-CT after treatment completion.

Lymph node biopsy and/or bone marrow for RNA extraction was obtained from samples when available at the following time points: At initial diagnosis, at relapse after frontline treatment and prior to initiating study treatment, and at relapse after initiation of study treatment when available. The SureSelect XT HS2 RNA System Library Prep Kit (Agilent) was used for cDNA preparation. Sequencing was performed using an Illumina NovaSeq 6000 10B 0.5 Flowcell at the WCM Genomics Resources Core Facility. Transcripts were quantified against Gencode hg38 genome using Salmon 1.10.0 REF1 [14]. Transcript-level abundances were summarized to the gene level via tximport (v1.32.0) and analyzed using DESeq2 (v1.44.0) [15]. Genes with a total count of < 10 across all samples were excluded from downstream analysis. Differential expression was modeled using a design formula based on EOT response. The DESeq function was executed using the standard pipeline (size factor estimation, dispersion estimation, and the Wald test). For visualization and clustering, including Principal Component Analysis (PCA), counts were transformed using the Variance Stabilizing Transformation (VST). Principal components were calculated based on the top 500 most variable genes. Multiple-testing correction was performed via the Benjamini-Hochberg method to control the False Discovery Rate (FDR). Lymphoma Microenvironment (LME) and Malignant Functional Process (MFP) classification were conducted using the BostonGene LME pipeline in Python 3. This approach utilizes functional expression signatures to categorize the stromal and immune landscapes based on transcriptomic signatures as previously described [12, 16]. All RNA-seq findings are presented as exploratory hypotheses intended to guide future larger-scale validation studies.

## Results

Enrollment was completed with nine patients (median age 58, range 45–71; 56% male). The cohort represented a high-risk population, with 89% relapsing < 1 year after frontline therapy (Table 1). All patients had prior anthracycline exposure for prior treatment of DLBCL.

Six patients were treated at DL1 (200 mg) and three at DL2 (300 mg). At DL2, one patient experienced a Grade 5 DLT (neutropenic sepsis) during cycle 1 of treatment, prompting a safety review of both DL1 and DL2. While subsequent DL2 patients did not strictly meet DLT criteria, frequent cycle delays (80%) and missed AZA doses (35%) due to persistent cytopenias led to a safety closure of DL2. In contrast, DL1 was well-tolerated with no DLTs, no cycle delays, and minimal missed AZA doses (4%) (Table 2). Based on these safety and feasibility data, DL1 (200 mg) was established as the RP2D. DL-1 (150 mg) did not warrant investigation based on tolerability of DL1 (200 mg).

**Fig. 2:**
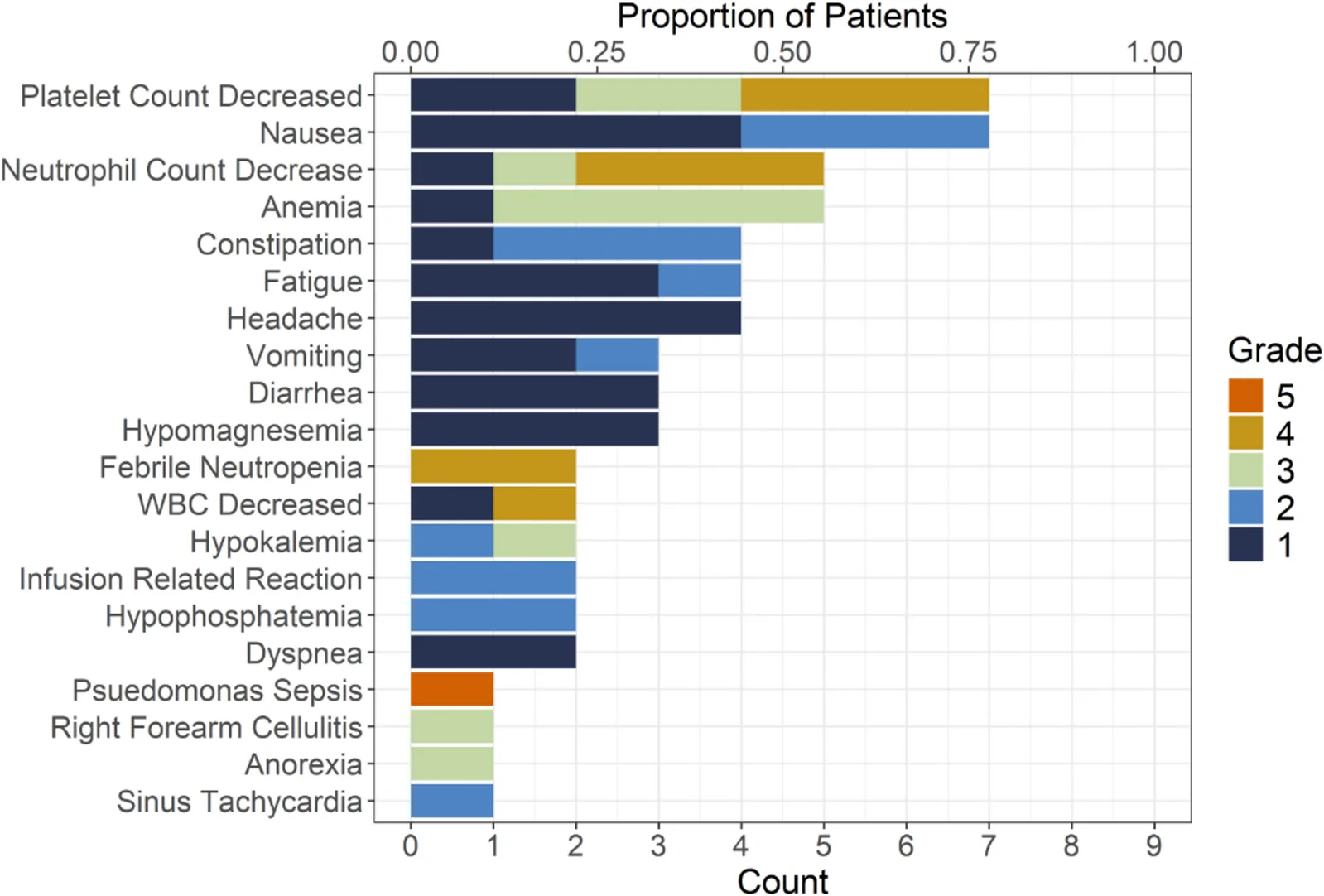
Top 20 adverse events ranked by frequency (All Doses)

The most frequent adverse events (AEs) across all grades included nausea (78%), thrombocytopenia (78%), and anemia/neutropenia (56%) (Fig. 2). Grade ≥ 3 hematologic toxicity was significantly more prevalent at DL2 compared to DL1, specifically neutropenia (100% vs. 17%) and neutropenic fever (67% vs. 0%). For a complete list of AEs and serious SAEs by dose level, see Appendix Tables 3, 4 and 5.

Among eight evaluable patients, excluding the patient with grade 5 neutropenic sepsis who passed away prior to response assessment, the overall response rate (ORR) was 50%, with all responders achieving complete remission (CR). Four patients (50%) successfully proceeded to ASCT with the other 4 patients not achieving adequate response to justify ASCT. All transplant-eligible patients achieved adequate peripheral blood stem cell (PBSC) collection defined as ≥ 3.0 × 10^6 CD34 cells/kg (median 4.08 × 10⁶ CD34 cells/kg). At a median follow-up of 15 months, the median progression-free survival (PFS) was 6 months (95% CI: 2.5–NR) and median overall survival (OS) was 35.5 months (95% CI: 9.5–NR). To date, all four patients proceeding to ASCT are alive with 3 of the 4 showing no evidence of relapsed lymphoma.

**Fig. 3:**
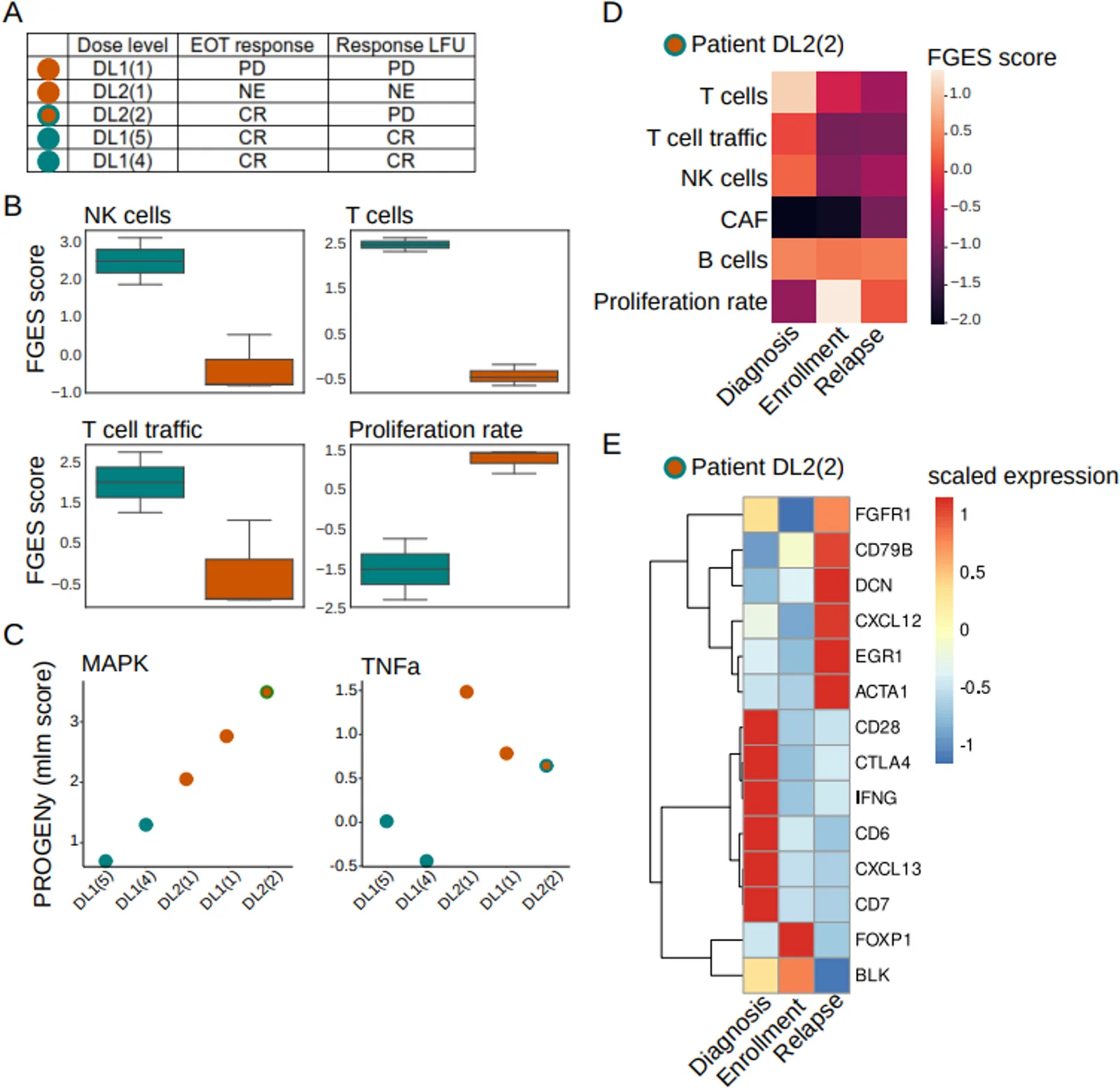
Transcriptional analysis of the lymphoma tissues of selected patients. **A**: Dose level and outcomes of analyzed patients. In orange nonresponders and in green responders at LFU. DL2(2) responded at EOT but eventually progressed at LFU. **B**. Transcriptional signatures of the indicated LME cell subtypes and pathways (FGES) in pre-treatment biopsies, comparing responders (green) and nonresponders (orange). **C**: MAPK and TNFA pathway activity (PROGENy) in responders (green) and nonresponders (orange). LME of patients not obtaining durable CR were found to have depletion of T-cells, NK cells, and T cell attractant cytokines **D**-**E**: Sequential analysis (LME signatures -**D**- and selected individual genes -**E**-) of lymphoma tissue from patient DL-2(2) obtained at diagnosis (before RCHOP), at enrollment (before trial) and at relapse. This patient showed initial response but subsequently relapse. The biopsy showed progressive depletion of T cell and NK cell signatures, and enrichment in fibrotic signatures

RNA-sequencing of pre-treatment biopsies (*n* = 5) revealed that patients with durable CRs exhibited lower tumor proliferation signatures and higher immune infiltration signatures (NK cells, T cells, and T-cell attractant cytokines) compared to non-responders or those who relapsed (Fig. 3). This pattern is consistent with the notion that DLBCL patients with immune-infiltrated LMEs exhibit a higher response to therapies, including epigenetic agents and immunochemotherapy [16]. An apparent exception to this pattern was a patient (DL2-2) with low immune infiltration signatures at enrolment that initially achieved CR; however, this patient subsequently relapsed showing even a more profound immune depletion LME signature (Fig. 3). Compared to the enrolment LME, the relapsed LME demonstrated progressive T-cell/NK-cell depletion and a marked increase in cancer-associated fibroblasts (CAF). Progression was further characterized by upregulation of *FGFR1* and downregulation of *IFNG*, indicating that azacitidine was unable to reverse an evolving immune-depleted fibrotic LME as a mechanism of resistance [17].

## Discussion

While therapeutic advances have improved outcomes for DLBCL, patients with relapsed/refractory (R/R) disease not meeting an indication for second-line CAR T-cell therapy still face suboptimal prognoses [18, 19]. We report the first phase I results investigating the addition of azacitidine (AZA) to R-ICE chemotherapy as an epigenetic sensitizing salvage strategy for transplant-eligible patients.

Our findings establish 200 mg (DL1) as the recommended phase 2 dose (RP2D). At this level, the regimen was well-tolerated with expected hematologic adverse events, no neutropenic fevers, and no dose-limiting toxicities. In contrast, 300 mg (DL2) resulted in excessive toxicity and R-ICE cycle delays, precluding its further use. Notably, our exploratory correlative studies suggests an immune-infiltrated LME signature in the pre-enrollment biopsies associated with a durable complete remission (CR) with this epigenetic-immunochemotherapy combination.

We did observed a 50% CR rate despite 89% of our cohort experiencing early relapse (< 1 year post-frontline therapy), a group historically associated with poor chemotherapy sensitivity and inferior post-ASCT outcomes [20, 21]. While CAR T-cell therapy has rightfully become the standard for early-relapsing DLBCL, its current regulatory approval does not extend to transplant eligible patients relapsing > 12 months after frontline treatment. However, the limited sample size of this dose-finding study precludes robust efficacy assessment, and its impact on the relapsed DLBCL treatment landscape is likely to be limited.

## Supplementary Information

Below is the link to the electronic supplementary material.

Supplementary Material 1

## Data Availability

Data supporting the findings of this study are available within the paper and its supplementary information. All other data is available upon request.
