# Supplementary material for: Phase I study of oral azacitidine plus salvage chemotherapy in relapsed/refractory diffuse large B-cell lymphoma
Source: Ann Hematol. 2026 Jun 22;105(9):405. doi: 10.1007/s00277-026-07125-7 (PMC13538234; doi:10.1007/s00277-026-07125-7)
Supplement: Supplementary file 1 [file 277_2026_7125_MOESM1_ESM.docx]

**Appendix Table 1: AZA Requalification Criteria and Dose Adjustments**

| **Timepoint** | **Event** | **Drug Action** |
| --- | --- | --- |
| Day -6 | ANC < 750 cells/mm^3^ unless thought to be related to bone marrow involvement per investigator assessment.  **AND/OR**  Platelet count < 75,000 cells/mm^3^ unless thought to be related to bone marrow involvement per investigator assessment | Do not initiate AZA and take patient off study as they are not a candidate per exclusion criteria |
| Day 1 (cycles 1-3) | ANC < 500 cells/mm^3^  **AND/OR**  Platelet count < 50,000 cells/mm^3^ | 1. Hold R-ICE chemotherapy. Reassess parameter no greater than one week later (physicians allowed to assess more frequently) 2. Resume R-ICE once subject meets both ANC and platelet count requalification criteria. |
|  | Non hematologic AE related to R-ICE | Delay, discontinue, or modify R-ICE at investigator discretion |
|  | Grade 3 or 4 Non hematologic AE related to AZA or other DLT | R-ICE can be started when AE resolved to grade 2 or less. |
| Day 8 (cycles 1-2) | ANC < 750 cells/mm^3^ with fever (temp > 38.5**°**C) | 1. Hold AZA and reassess ANC no greater than one week later. Physicians are allowed to assess more frequently. AZA can be restarted once ANC is ≥ 500 cells/mm^3^ and fever has resolved. 2. If requalification criteria is not met by day 15 then AZA should be suspended the remainder of the current cycle and reduce by one dose level at the next cycle according to table 2. |
| Day 15 (cycles 1-2) | ANC < 500 cells/mm^3^  **AND/OR**  Platelet count < 25,000 cells/mm^3^ | 1. Hold AZA and reassess qualification criteria as soon as 24 hours later and no later than 72 hours later. AZA can be restarted once requalification criteria has been met. If at 72 hours patient does not meet qualification criteria, then hold AZA until next cycle and reduce by one dose level according to table 2. 2. If this was the first occurrence of holding AZA because of hematologic qualification criteria then restart AZA at the same dose level according to requalification criteria. For subsequent occurrences reduce by one dose level once requalification are met according to table 2. |
| Unscheduled assessment | DLT | Hold AZA until recovery to ≤ grade 2, then restart at next lower dose level. If there is no lower dose level then do not restart AZA and continue with R-ICE alone. AZA can be permanently stopped by investigator with any DLT. |

**Appendix Table 2: AZA Dose Level Reductions**

| Dose level | Starting dose | Dose reduction No. 1 | Dose reduction No. 2 | Dose reduction No. 3 | Dose reduction No. 4 |
| --- | --- | --- | --- | --- | --- |
| -2 | 100 mg QD | 100 mg QOD | None | None | None |
| -1 | 150 mg QD | 100 mg QD | 100 mg QOD | None | None |
| 1 | 200 mg QD | 150 mg QD | 100 mg | 100 mg QOD | None |
| 2 | 300 mg QD | 200 mg QD | 150 mg | 100 mg QD | 100 mg QOD |

| **Appendix Table 3. All Adverse Events in Descending Frequency within System Organ Class for CC-486 Dose Level 200mg** | | | | | |
| --- | --- | --- | --- | --- | --- |
| **Adverse Event** | **N = 6** | | | | |
|  | **Grade 1** | **Grade 2** | **Grade 3** | **Grade 4** | **Overall** |
|  | **n (%)** | **n (%)** | **n (%)** | **n (%)** |  |
| **Gastrointestinal** | 2 (33) | 4 (67) | — | — | 6 (100) |
| Nausea | 3 (50) | 1 (17) | — | — | 4 (67) |
| Vomiting | 2 (33) | 1 (17) | — | — | 3 (50) |
| Constipation | — | 2 (33) | — | — | 2 (33) |
| Decreased Appetite | 1 (17) | — | — | — | 1 (17) |
| Diarrhea | 1 (17) | — | — | — | 1 (17) |
| **Investigations** | 2 (33) | — | 1 (17) | 1 (17) | 4 (67) |
| Platelet Count Decreased | 2 (33) | — | 1 (17) | 1 (17) | 4 (67) |
| Neutrophil Count Decrease | 1 (17) | — | 1 (17) | — | 2 (33) |
| WBC Decreased | 1 (17) | — | — | 1 (17) | 2 (33) |
| Alkaline Phosphatase Increased | 1 (17) | — | — | — | 1 (17) |
| Alt Increased | 1 (17) | — | — | — | 1 (17) |
| Ast Increased | 1 (17) | — | — | — | 1 (17) |
| **Metabolism** | 2 (33) | 2 (33) | — | — | 4 (67) |
| Creatinine Increased | 1 (17) | — | — | — | 1 (17) |
| Dehydration | — | 1 (17) | — | — | 1 (17) |
| Hyperglycemia | 1 (17) | — | — | — | 1 (17) |
| Hypokalemia | — | 1 (17) | — | — | 1 (17) |
| Hypomagnesemia | 1 (17) | — | — | — | 1 (17) |
| Hypophosphatemia | — | 1 (17) | — | — | 1 (17) |
| **Musculoskeletal** | 2 (33) | 1 (17) | 1 (17) | — | 4 (67) |
| Arthralgia | 1 (17) | — | — | — | 1 (17) |
| Back Pain | — | 1 (17) | — | — | 1 (17) |
| Bilateral Hip Pain | 1 (17) | — | — | — | 1 (17) |
| Bone Pain | 1 (17) | — | — | — | 1 (17) |
| Worsening Pain | — | — | 1 (17) | — | 1 (17) |
| **Blood** | 1 (17) | — | 1 (17) | 1 (17) | 3 (50) |
| Anemia | 1 (17) | — | 2 (33) | — | 3 (50) |
| Lymphocyte Count Decrease | — | — | — | 1 (17) | 1 (17) |
| **Nervous** | 3 (50) | — | — | — | 3 (50) |
| Headache | 2 (33) | — | — | — | 2 (33) |
| Dysgeusia | 1 (17) | — | — | — | 1 (17) |
| **Respiratory** | 2 (33) | 1 (17) | — | — | 3 (50) |
| Cough | 1 (17) | — | — | — | 1 (17) |
| Nasal Congestion | — | 1 (17) | — | — | 1 (17) |
| Sneezing | 1 (17) | — | — | — | 1 (17) |
| Sore Throat | 1 (17) | — | — | — | 1 (17) |
| **Eye** | 1 (17) | 1 (17) | — | — | 2 (33) |
| Conjunctivitis Left Eye | — | 1 (17) | — | — | 1 (17) |
| Watering Eyes | 1 (17) | — | — | — | 1 (17) |
| **General** | 1 (17) | 1 (17) | — | — | 2 (33) |
| Fatigue | 2 (33) | — | — | — | 2 (33) |
| Edema Limbs | 1 (17) | — | — | — | 1 (17) |
| Infusion Related Reaction | — | 1 (17) | — | — | 1 (17) |
| **Renal** | 2 (33) | — | — | — | 2 (33) |
| Dysuria | 1 (17) | — | — | — | 1 (17) |
| Urinary Frequency | 1 (17) | — | — | — | 1 (17) |
| **Cardiac** | — | 1 (17) | — | — | 1 (17) |
| Tachycardia | — | 1 (17) | — | — | 1 (17) |
| **Psychiatric** | — | — | 1 (17) | — | 1 (17) |
| Worsening Depression | — | — | 1 (17) | — | 1 (17) |
| **Skin** | 1 (17) | — | — | — | 1 (17) |
| Bullous Dermatitis | 1 (17) | — | — | — | 1 (17) |

| **Appendix Table 3a. All Related Adverse Events in Descending Frequency within System Organ Class for CC-486 Dose Level 200mg** | | | | | |
| --- | --- | --- | --- | --- | --- |
| **Adverse Event** | **N = 6** | | | | |
|  | **Grade 1 n (%)** | **Grade 2 n (%)** | **Grade 3 n (%)** | **Grade 4 n (%)** | **Overall** |
| **Gastrointestinal** | 2 (33) | 3 (50) | — | — | 5 (83) |
| Nausea | 3 (50) | 1 (17) | — | — | 4 (67) |
| Vomiting | 2 (33) | 1 (17) | — | — | 3 (50) |
| Constipation | — | 1 (17) | — | — | 1 (17) |
| Decreased Appetite | 1 (17) | — | — | — | 1 (17) |
| Diarrhea | 1 (17) | — | — | — | 1 (17) |
| **Investigations** | 2 (33) | — | 1 (17) | 1 (17) | 4 (67) |
| Platelet Count Decreased | 2 (33) | — | 1 (17) | 1 (17) | 4 (67) |
| Neutrophil Count Decrease | 1 (17) | — | 1 (17) | — | 2 (33) |
| WBC Decreased | 1 (17) | — | — | 1 (17) | 2 (33) |
| Alt Increased | 1 (17) | — | — | — | 1 (17) |
| Ast Increased | 1 (17) | — | — | — | 1 (17) |
| **Blood** | 1 (17) | — | 2 (33) | — | 3 (50) |
| Anemia | 1 (17) | — | 2 (33) | — | 3 (50) |
| **Metabolism** | 1 (17) | 2 (33) | — | — | 3 (50) |
| Creatinine Increased | 1 (17) | — | — | — | 1 (17) |
| Dehydration | — | 1 (17) | — | — | 1 (17) |
| Hypokalemia | — | 1 (17) | — | — | 1 (17) |
| Hypomagnesemia | 1 (17) | — | — | — | 1 (17) |
| Hypophosphatemia | — | 1 (17) | — | — | 1 (17) |
| **Nervous** | 3 (50) | — | — | — | 3 (50) |
| Headache | 2 (33) | — | — | — | 2 (33) |
| Dysgeusia | 1 (17) | — | — | — | 1 (17) |
| **General** | 1 (17) | 1 (17) | — | — | 2 (33) |
| Fatigue | 1 (17) | — | — | — | 1 (17) |
| Infusion Related Reaction | — | 1 (17) | — | — | 1 (17) |
| **Musculoskeletal** | 2 (33) | — | — | — | 2 (33) |
| Arthralgia | 1 (17) | — | — | — | 1 (17) |
| Bone Pain | 1 (17) | — | — | — | 1 (17) |
| **Renal** | 1 (17) | — | — | — | 1 (17) |
| Urinary Frequency | 1 (17) | — | — | — | 1 (17) |

| **Appendix Table 4. All Adverse Events in Descending Frequency within System Organ Class for CC-486 Dose Level 300mg** | | | | | | |
| --- | --- | --- | --- | --- | --- | --- |
| **Adverse Event** | **N = 3** | | | | | |
|  | **Grade 1** | **Grade 2** | **Grade 3** | **Grade 4** | **Grade 5** | **Overall** |
|  | **n (%)** | **n (%)** | **n (%)** | **n (%)** | **n (%)** |  |
| **Blood** | — | — | 1 (33) | 2 (67) | — | 3 (100) |
| Anemia | — | — | 2 (67) | — | — | 2 (67) |
| Febrile Neutropenia | — | — | — | 2 (67) | — | 2 (67) |
| **Gastrointestinal** | — | 3 (100) | — | — | — | 3 (100) |
| Nausea | 1 (33) | 2 (67) | — | — | — | 3 (100) |
| Constipation | 1 (33) | 1 (33) | — | — | — | 2 (67) |
| Diarrhea | 2 (67) | — | — | — | — | 2 (67) |
| Mucositis Oral | — | 1 (33) | — | — | — | 1 (33) |
| **Investigations** | — | — | — | 3 (100) | — | 3 (100) |
| Neutrophil Count Decrease | — | — | — | 3 (100) | — | 3 (100) |
| Platelet Count Decreased | — | — | 1 (33) | 2 (67) | — | 3 (100) |
| **Metabolism** | — | 1 (33) | 2 (67) | — | — | 3 (100) |
| Hypomagnesemia | 2 (67) | — | — | — | — | 2 (67) |
| Anorexia | — | — | 1 (33) | — | — | 1 (33) |
| Hypokalemia | — | — | 1 (33) | — | — | 1 (33) |
| Hypophosphatemia | — | 1 (33) | — | — | — | 1 (33) |
| **Respiratory** | 2 (67) | 1 (33) | — | — | — | 3 (100) |
| Dyspnea | 2 (67) | — | — | — | — | 2 (67) |
| Pulmonary Edema | — | 1 (33) | — | — | — | 1 (33) |
| Sinus Pain | 1 (33) | — | — | — | — | 1 (33) |
| **General** | 1 (33) | 1 (33) | — | — | — | 2 (67) |
| Fatigue | 1 (33) | 1 (33) | — | — | — | 2 (67) |
| Chills | 1 (33) | — | — | — | — | 1 (33) |
| Fever | 1 (33) | — | — | — | — | 1 (33) |
| Infusion Related Reaction | — | 1 (33) | — | — | — | 1 (33) |
| **Infections** | — | — | 1 (33) | — | 1 (33) | 2 (67) |
| Psuedomonas Sepsis | — | — | — | — | 1 (33) | 1 (33) |
| Right Forearm Cellulitis | — | — | 1 (33) | — | — | 1 (33) |
| **Nervous** | 2 (67) | — | — | — | — | 2 (67) |
| Headache | 2 (67) | — | — | — | — | 2 (67) |
| **Renal** | 1 (33) | 1 (33) | — | — | — | 2 (67) |
| Bladder Spasm | — | 1 (33) | — | — | — | 1 (33) |
| Urinary Incontinence | 1 (33) | — | — | — | — | 1 (33) |
| **Skin** | 1 (33) | 1 (33) | — | — | — | 2 (67) |
| Alopecia | — | 1 (33) | — | — | — | 1 (33) |
| Hyperpigmentation | 1 (33) | — | — | — | — | 1 (33) |
| Nose Abrasion | 1 (33) | — | — | — | — | 1 (33) |
| Petechiae Bilateral Upper Extremity | 1 (33) | — | — | — | — | 1 (33) |
| **Cardiac** | — | 1 (33) | — | — | — | 1 (33) |
| Sinus Tachycardia | — | 1 (33) | — | — | — | 1 (33) |
| **Injury** | — | 1 (33) | — | — | — | 1 (33) |
| Bruising Right Forearm | 1 (33) | — | — | — | — | 1 (33) |
| Fall | — | 1 (33) | — | — | — | 1 (33) |
| Right Forearm Abrasion | 1 (33) | — | — | — | — | 1 (33) |
| **Musculoskeletal** | — | 1 (33) | — | — | — | 1 (33) |
| Generalized Muscle Weakness | — | 1 (33) | — | — | — | 1 (33) |
| **Psychiatric** | — | 1 (33) | — | — | — | 1 (33) |
| Altered Mental Status | — | 1 (33) | — | — | — | 1 (33) |
| **Vascular** | — | 1 (33) | — | — | — | 1 (33) |
| Hypotension | — | 1 (33) | — | — | — | 1 (33) |

| **Appendix Table 4a. All Related Adverse Events in Descending Frequency within System Organ Class for CC-486 Dose Level 300mg** | | | | | | |
| --- | --- | --- | --- | --- | --- | --- |
| **Adverse Event** | **N = 3** | | | | | |
|  | **Grade 1 n (%)** | **Grade 2 n (%)** | **Grade 3 n (%)** | **Grade 4 n (%)** | **Grade 5 n (%)** | **Overall** |
| **Blood** | — | — | 1 (33) | 2 (67) | — | 3 (100) |
| Anemia | — | — | 2 (67) | — | — | 2 (67) |
| Febrile Neutropenia | — | — | — | 2 (67) | — | 2 (67) |
| **Gastrointestinal** | — | 3 (100) | — | — | — | 3 (100) |
| Nausea | 1 (33) | 2 (67) | — | — | — | 3 (100) |
| Constipation | 1 (33) | 1 (33) | — | — | — | 2 (67) |
| Diarrhea | 2 (67) | — | — | — | — | 2 (67) |
| Mucositis Oral | — | 1 (33) | — | — | — | 1 (33) |
| **Investigations** | — | — | — | 3 (100) | — | 3 (100) |
| Neutrophil Count Decrease | — | — | — | 3 (100) | — | 3 (100) |
| Platelet Count Decreased | — | — | 1 (33) | 2 (67) | — | 3 (100) |
| **Metabolism** | — | 1 (33) | 2 (67) | — | — | 3 (100) |
| Hypomagnesemia | 2 (67) | — | — | — | — | 2 (67) |
| Anorexia | — | — | 1 (33) | — | — | 1 (33) |
| Hypokalemia | — | — | 1 (33) | — | — | 1 (33) |
| Hypophosphatemia | — | 1 (33) | — | — | — | 1 (33) |
| **General** | 1 (33) | 1 (33) | — | — | — | 2 (67) |
| Fatigue | 1 (33) | 1 (33) | — | — | — | 2 (67) |
| Chills | 1 (33) | — | — | — | — | 1 (33) |
| Fever | 1 (33) | — | — | — | — | 1 (33) |
| **Infections** | — | — | 1 (33) | — | 1 (33) | 2 (67) |
| Psuedomonas Sepsis | — | — | — | — | 1 (33) | 1 (33) |
| Right Forearm Cellulitis | — | — | 1 (33) | — | — | 1 (33) |
| **Respiratory** | 2 (67) | — | — | — | — | 2 (67) |
| Dyspnea | 2 (67) | — | — | — | — | 2 (67) |
| **Skin** | 1 (33) | 1 (33) | — | — | — | 2 (67) |
| Alopecia | — | 1 (33) | — | — | — | 1 (33) |
| Hyperpigmentation | 1 (33) | — | — | — | — | 1 (33) |
| Nose Abrasion | 1 (33) | — | — | — | — | 1 (33) |
| Petechiae Bilateral Upper Extremity | 1 (33) | — | — | — | — | 1 (33) |
| **Cardiac** | — | 1 (33) | — | — | — | 1 (33) |
| Sinus Tachycardia | — | 1 (33) | — | — | — | 1 (33) |
| **Injury** | — | 1 (33) | — | — | — | 1 (33) |
| Fall | — | 1 (33) | — | — | — | 1 (33) |
| **Musculoskeletal** | — | 1 (33) | — | — | — | 1 (33) |
| Generalized Muscle Weakness | — | 1 (33) | — | — | — | 1 (33) |
| **Nervous** | 1 (33) | — | — | — | — | 1 (33) |
| Headache | 1 (33) | — | — | — | — | 1 (33) |
| **Renal** | — | 1 (33) | — | — | — | 1 (33) |
| Bladder Spasm | — | 1 (33) | — | — | — | 1 (33) |
| **Vascular** | — | 1 (33) | — | — | — | 1 (33) |
| Hypotension | — | 1 (33) | — | — | — | 1 (33) |

| **Appendix Table 5. All Related Serious Adverse Events in Descending Frequency within System Organ Class for CC-486 Dose Level 300mg** | | |
| --- | --- | --- |
| **Adverse Event** | **N = 3** | |
|  | **Grade 4 n (%)** | **Overall** |
| **Blood** | 2 (67) | 2 (67) |
| Febrile Neutropenia | 2 (67) | 2 (67) |
| **Infections** | 1 (33) | 1 (33) |
| Psuedomonas Sepsis | 1 (33) | 1 (33) |
| **Investigations** | 1 (33) | 1 (33) |
| Neutrophil Count Decrease | 1 (33) | 1 (33) |

**NOTE:** All SAE were related; All SAE were in 300mg dose level
